# German dentists’ websites on periodontitis have low quality of information

**DOI:** 10.1186/s12911-017-0511-8

**Published:** 2017-08-02

**Authors:** Falk Schwendicke, Jörg Stange, Claudia Stange, Christian Graetz

**Affiliations:** 1 Department of Operative and Preventive Dentistry, Charité – Universitätsmedizin Berlin, Aßmannshauser Str. 4-6, 14199 Berlin, Germany; 2 Stange Dental, Wilhelmstraße 1, 25436 Tornesch, Germany; 3 Clinic of Conservative Dentistry and Periodontology, University Medical Center Schleswig-Holstein, Arnold-Heller-Str. 3 (Haus 26), 24105 Kiel, Germany

**Keywords:** Decision making, Evidence-based dentistry, Health services research, Internet, Periodontitis, Public health, Shared decision making

## Abstract

**Background:**

The internet is an increasingly relevant source of health information. We aimed to assess the quality of German dentists’ websites on periodontitis, hypothesizing that it was significantly associated with a number of practice-specific parameters.

**Methods:**

We searched four electronic search engines and included pages which were freely accessible, posted by a dental practice in Germany, and mentioned periodontal disease/therapy. Websites were assessed for (1) technical and functional aspects, (2) generic quality and risk of bias, (3) disease-specific information. For 1 and 2, validated tools (LIDA/DISCERN) were used for assessment. For 3, we developed a criterion catalogue encompassing items on etiologic and prognostic factors for periodontitis, the diagnostic and treatment process, and the generic chance of tooth retention in periodontitis patients. Inter- and intra-rater reliabilities were largely moderate. Generalized linear modeling was used to assess the association between the information quality (measured as % of maximally available scores) and practice-specific characteristics.

**Results:**

Seventy-one websites were included. Technical and functional aspects were reported in significantly higher quality (median: 71%, 25/75th percentiles: 67/79%) than all other aspects (*p* < 0.05). Generic risk of bias and most disease-specific aspects showed significantly lower reporting quality (median range was 0–40%), with poorest reporting for prognostic factors (9;0/27%), diagnostic process (0;0/33%) and chances of tooth retention (0;0/2%). We found none of the practice-specific parameters to have significant impact on the overall quality of the websites.

**Conclusions:**

Most German dentists’ websites on periodontitis are not fully trustworthy and relevant information are not or insufficiently considered. There is great need to improve the information quality from such websites at least with regards to periodontitis.

## Background

Globally, around 50% of the population has access to the internet; this share increases to 90% or above in most industrialized countries [1]. Of those with access, up to 55% have searched the internet once or more times for health-related information [2], usually starting their search via a search engine [3]. For dental health-related information, a number of possible sources are available; dental practice websites, public or education websites, websites from dental material manufacturers, laboratories etc. Dental patients are likely to obtain information from websites of either their dentist or, in case they do not have a regular dentist, by screening through various sites on the search for both information and a professional who could deliver care. Moreover, the information provided on dentists’ websites might reflect on what dentists inform their patients on in the chair, for example with regards to the etiology, prevention, therapy and maintenance of a specific dental disease (like caries or periodontitis).

The quality of a website can be assessed threefold; technically, with regards to its “generic” risk of bias using broad spun criteria for assessment, and with regards to the specific disease-related information provided. A large number of tools is available to assess the technical and functional aspects and the generic risk of bias. The LIDA and the DISCERN instrument, for example, allow users to systematically evaluate a website’s accessibility, usability, reliability and generic quality of information [4, 5]. Such tools have been successfully applied to evaluate the quality of information of websites on surgery or endocrinology [6, 7]. Similar generic instruments have been applied to judge the quality of information of websites on periodontitis, assessing for example the currency of the website and the justifiability and transparency of the information provided [8], finding the quality of information provided to be rather poor.

So far, it has not been assessed if dentists’ websites allow dental patients or the public to gain high quality disease-specific information on periodontitis. For patients, for example, it is relevant to understand how periodontitis develops and which prognostic factors are known, as this might facilitate a prevention-focused behavior. Moreover, patients might want to know how the disease is diagnosed and treated, how likely it is to lose a tooth due to periodontitis, and if a tooth replacement using, for example, an implant, shows better or worse success and survival than a periodontally affected tooth. On all these aspects, dentists should be knowledgeable; one could thus expect an informative website to comprehensively cover these issues.

The present study assessed the quality of German dentists’ websites on periodontitis. Our aim was to evaluate both the technical and generic quality but also the quality of disease-specific information. We hypothesized that the overall information quality was significantly associated with a number of practice-specific parameters like the practice type, setting, the owner’s age and his/her degree of specialization towards periodontology.

## Methods

### Sample size calculation

Our main outcome parameter was the overall quality of a website, indicated on a continuous scale (% of maximally possible scores summed from ordinal scaled items, see below). We planned to assess how a total of four independent (practice-specific) parameters impacted on this outcome parameter using generalized linear modelling. The minimum required number of websites to be included on our analysis (that is, the sample size) for such regression analysis was estimated assuming the level of significance α = 0.05, the number of predictors in the model to be 4, the anticipated effect size to be 0.2 (that is, moderate), and the desired statistical power to be β = 0.80. The calculation was performed using G*Power 3.1.9.2 (University of Dusseldorf, Germany). A minimum of 64 websites was estimated to be needed for this study (F = 2.52; df = 4, expected power 81%). Note that we did not include any corrections for multiple testing here, as sample size was estimated based on the primary outcome (overall quality) only. The performed regression analyses for the sub-totals of quality in different domains (see below) might thus have been under-powered and should be considered as exploratory in nature.

### Search

We searched four electronic search engines (Google Germany, Bing, Yahoo, Ask.com) on the 24th to 27th June 2016 using the keyword “Parodontitis” (German for periodontitis) and “Parodontose” (the latter being the plain language term used by lay people). For the search a computer connected to the Internet in Germany was used after clearing cookies and the browser history, without modifying the default setting of the search engines. A total of 568 websites were displayed after removal of duplicates (Google: 241, Bing: 375, Yahoo: 437, Ask.com: 29). Note the displayed number of sites was much lower than the number of identified sites (on Google, for example, 130,000 sites were identified); however, the search machines only displayed the “most relevant” sites, allowing to display all identified sites only if desired at the end of the search. We did not expand the search beyond the displayed number of sites.

Websites were excluded at this stage if they clearly contained irrelevant information, leaving 558 websites, which were potentially eligible. These were screened full-text by one examiner (JS). We applied the following inclusion criteria: (1) Main page freely accessible, (2) in German, (3) posted by a dental practice or practice cooperation in Germany, (4) mentioning periodontal disease and/or therapy anywhere on the site. We excluded websites from or associated with dental laboratories or supply/materials companies, professional forums or blogs, dental regulatory or research or otherwise public bodies.

### Outcomes

Websites were assessed for their quality in a number of domains. The first two domains were built using established and validated generic instruments, as described below:

- (1) Technical and functional aspects
- (2) Generic quality and risk of bias

The third domain focused on the quality of disease-specific information. We assumed comprehensive and high quality information could be expected to be provided on a fully informative website to allow patients or other stakeholders to inform themselves and make conscious and informed decisions, for example regarding tooth retention or removal. This third domain was structured along five sub-domains, regarding information provided on

- (3.1) Etiologic factors for periodontitis
- (3.2) Prognostic factors for periodontitis
- (3.3) The diagnostic process encompassing periodontal diagnosis and treatment planning
- (3.4) The treatment process stages
- (3.5) The generic chance of tooth retention in periodontitis patients and a comparative estimate on implant success and survival, allowing the patient to make an informed decision, if needed, on to whether to attempt tooth retention or removal and replacement.

For the first two domains, validated tools were modified according to the purpose of this study (the modification included omission of questions deemed irrelevant or redundant with those in the disease-specific domain). These tools, LIDA 1.2 and DISCERN, have been described in detail elsewhere [4, 5] and have been used in similar studies as described above. LIDA is an application measuring the accessibility, usability (including clarity, consistency, functionality, engagability) and reliability (currency, conflict of interest, content production) of websites. Items are scored on an ordinal scale as 0 (never), 1 (sometimes), 2 (mostly) and 3 (always). Item scores are then summed up in a compound score and the % of maximally possible score sums is calculated. Score sums >90% represent good results and <50% represent poor results.

The DISCERN tool is a user guidance toolkit allowing to judge the generic quality and risk of bias of health information websites with regards to treatment choices, looking at reliability (trust in the information that is based on source of information) and quality of information (information on treatment alternatives). Scores are given on an ordinal scale from (0 no) to 5 (yes). As there was overlap between both instruments, conflict of interest in the LIDA scale was assessed jointly with reliability items of DISCERN. For ease of data extraction and analysis, we eventually scored items as 0 (never/no), 1 (sometimes/partially) and 2 (mostly/always/yes) in both domains. The used guiding questions are given together with the results in Tables 1, 2.

**Table 1:** Technical and functional aspects (domain 1). Sub-domains with a range of items were assessed. Scores between 0 and 2 were used

Sub-domain	Item	Median (25th/75th percentiles; min-max)
Accessibility	Does it work on a range of browsers?	2 (1/2; 0–2)
Accessibility	Is it fully free to use?	2 (2/2; 0–2)
Usability	Is it easy to navigate?	2 (1/2; 0–2)
Clarity	Are all links working?	2 (2/2; 0–2)
Clarity	Is it easy to understand?	2 (2/2; 0–2)
Clarity	Is the layout of the main block of information clear and readable?	2 (2/2; 0–2)
Functionality	Does it have an effective search function?	0 (0/0, 0–0)
Functionality	Does it work without plugins?	2 (1/2; 0–2)
Functionality	Does the design minimize the cognitive overhead?	2 (1/2; 0–2)
Incapability	Is it interactive?	0 (0/0; 0–2)
Currency?	Is it current?	2 (1/2; 0–2)
Currency	Does the site respond to recent events?	1 (0/1; 0–2)

**Table 2:** Generic quality and risk of bias (domain 2). Sub-domains with a range of items were assessed. Scores between 0 and 2 were used

Sub-domain	Item	Median (25th/75th percentiles, min-max)
Reliability	Are the aims clear?	2 (2/2; 0–2)
Reliability	Is it clear who pays for it?	1 (1/1; 0–2)
Reliability	Is there a declaration of the objectives of the people who run the site?	2 (2/2; 0–2)
Reliability	Is it clear who runs the site?	0 (0/2; 0–2)
Reliability	Is it current?	2 (2/2; 0–2)
Reliability	Is it clear what information sources were used?	0 (0/1; 0–2)
Reliability	Is it clear when the information sources were produced?	0 (0/0; 0–2)
Reliability	Is it balanced and unbiased?	2 (2/2; 0–2)
Reliability	Does it provide details of additional sources?	0 (0/1; 0–2)
Reliability	Does it refer to areas of uncertainty?	0 (0/1; 0–2)
Quality	Does it describe how each treatment works?	1 (0/1; 0–2)
Quality	Does it describe the benefit of each treatment?	1 (0/1; 0–2)
Quality	Does it describe the risk of each treatment?	0 (0/0; 0–2)
Quality	Does it describe what would happen if no treatment is used?	0 (0/0; 0–2)
Quality	Does it describe how the choice of treatment affect quality of life?	0 (0/1; 0–2)
Quality	Is it clear that there may be more than one possible treatment?	1 (0/1; 0–2)
Quality	Does it provide support for shared decision making?	1 (1/2; 0–2)

Questions on disease-specific information were developed jointly by the authors, building on quality guidelines from Switzerland [9]. The guideline gives instructions on how to perform periodontal diagnosis, treatment planning and therapy. In detail, the authors describe two objectives for periodontal diagnostics: First, it should assist to identify persons in need for treatment (diseased individuals or those with increased risk of periodontitis), and in case of persons in need for treatment, diagnostics should provide all information to develop an individual treatment plan as well as to control the success of the therapy. For re-evaluation after APT, but also at each re-evaluation during SPT, the patient is categorized as A+, A, B, C. These are defined in five domains; diagnostics, non-surgical therapy, surgical therapy, supportive periodontal therapy and compliance. For example, A+ is defined as no PPD > 4 mm with BOP, minimal BOP, no furcation involvement with signs of inflammation, no hard or soft deposits, optimal occlusion and aesthetics, non- or former smoking status. In contrast, for example, category C is defined by suppuration, recurrent abscess formation, neglect of oral hygiene, generalized BOP and progressive loss of attachment in several sites with persistence of function-impairing occlusal disturbances. Using these criteria, practitioners can assess the success of their therapy at each periodontal treatment phase and can also identify areas where improvement is needed. Again, a 3-point ordinal scale was used to answer the questions. Questions can be found in Table 3.

**Table 3:** Periodontitis-specific aspects (domain 3). Sub-domains with a range of items were assessed. Scores between 0 and 2 were used

Sub-domain	Item	Median (25th/75th percentiles, min-max)
3.1 Etiologic factors	Plaque as main cause	2 (0/2; 0–2)
	Multifactorial etiology	0 (0/0; 0–2)
	Attachment loss as main sign	2 (0/2; 0–2)
	Widespread disease	0 (0/0; 0–2)
	Frequent reason for tooth loss	0 (0/0; 0–2)
3.2 Prognostic factors	Smoking	0 (0/2; 0–2)
	Oral hygiene	0 (0/2; 0–2)
	Age	0 (0/2; 0–2)
	Diabetes	0 (0/2; 0–2)
	Bone loss	0 (0/0; 0–2)
	Tooth mobility	0 (0/0; 0–2)
	Furcation involvement	0 (0/0; 0–2)
	Probing depths	0 (0/0; 0–2)
	Parafunctions	0 (0/0; 0–0)
	Endodontic treatment	0 (0/0; 0–0)
	Compliance	0 (0/2; 0–2)
3.3 Diagnostic process	History	0 (0/2; 0–2)
	Dental and periodontal status	0 (0/0; 0–2)
	Radiographs	0 (0/0; 0–2)
3.4 Treatment stages	Initial (hygiene) phase	0 (0/2; 0–2)
	Active periodontal treatment	0 (0/2; 0–2)
	Supportive periodontal treatment	2 (0/2; 0–2)
3.5 Tooth retention or removal	Chance for tooth retention	0 (0/0; 0–0)
	Comparison with implants	0 (0/0; 0–1)

From all three domains, an overall quality score was eventually calculated as the sum of all achieved scores per the maximally possible score sum (in %). This was our primary outcome parameter. The secondary outcomes were the quality in each domain and sub-domain, again measured as % of achieved sum scores per all possible scores (in %).

As discussed, we aimed to assess if information quality was associated with one or more of the following practice-specific parameters: (1) Practice type, as single owner practice versus group/cooperation owner, (2) place of the practice, as rural versus city >5000 inhabitants, (3) age of the practice owner or lead dentist, as <50 years versus 50 years or older (extracted from available CVs), and specialization or specific interest in periodontology, indicated by being a member of the German association of Periodontology (DGPARO). For 16 websites, not all of these variables could be recorded; these were handled as randomly missing and excluded from this analysis.

### Data extraction and reliability

One researcher (JS) extracted the data on all websites. To estimate intra-rater reliability, the same researcher repeated the extraction process on a random subset of 20 websites 2 weeks after the initial assessment without information on the first extraction. To estimate inter-rater reliability, a second researcher (CS) repeated the data extraction on the same set of websites. The intra- and inter-rater reliability were expressed as kappa coefficients for single items and intra-class correlation coefficients for domain sums. The latter were 0.95/0.54 for domain 1, 0.77/0.32 for domain 2, 0.87/0.61 for domain 3.1, 0.89/0.87 for domain 3.2, 0.78/0.19 for domain 3.3, 0.66/0.72 for domain 3.4 and 1.0/1.0 for domain 3.5. Overall intra- and inter-rater reliability were 0.92 and 0.62, respectively. Reliability of assessed practice-specific parameters ranged between kappa = 0.8–1.0. Reliability estimation was performed prior to conducting the main study, as this allowed to recalibrate questions after discussing disagreement. No second reliability assessment was performed.

### Statistical analysis

Median, quartiles and ranges were used for descriptive statistical analysis. Statistical differences in quality of reporting between domains was tested using Wilcoxon’s test. Generalized linear modeling was used to assess the association between the overall quality (in %) or the domain quality, and practice-specific characteristics. Multivariable analysis with simultaneous entering of covariates was performed and main effects tested; no interaction terms were used as this usually requires additional model development, with increasing risk of alpha-inflation. Statistical significance was assumed if *p* < 0.05.

## Results

**Fig. 1:**
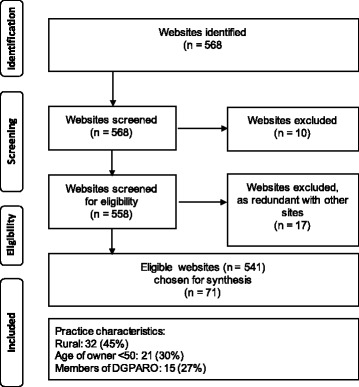
Flowchart of the search

From the 558 potentially eligible websites, 17 were found ineligible. The remaining 541 websites were alphabetically ordered, and every eighth website included in the present study, yielding a total sample of 71 websites (Fig. 1).

**Fig. 2:**
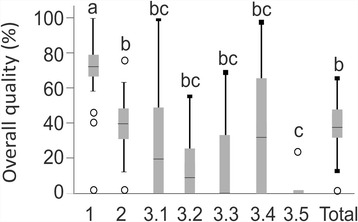
Information quality in different domains, as relative % per maximal possible sum score. Significant differences are indicated by different superscript letters (*p* < 0.05; Wilcoxon). Box and line: 25th/75th percentiles and median; whiskers: range, circles: outliers

Website quality differed significantly according to the domains evaluated (Fig. 2). The technical and functional aspects were found to be reported in significantly higher quality (median: 71%, 25/75th percentiles: 67/79%) than all other aspects (*p* < 0.05/Wilcoxon). Generic risk of bias and most disease-specific sub-domains showed significantly lower reporting quality (median range was 0–40%), with the poorest reporting in the sub-domains of prognostic factors (9; 0/27%), diagnostic process (0; 0/33%) and tooth retention versus removal (0; 0/2%).

Within each domain, differences were limited, with nearly all items in the domain 1 (technical and functional aspects) showing high quality (Table 1). The domain 2 (generic quality and risk of bias) showed mixed quality (Table 2), while those in the domain 3 (disease-specific information) showed low quality for nearly all items (Table 3).

Using multivariable regression analysis, we found none of the practice-specific parameters to have significant impact on the overall quality of the websites (Table 4). Only for the sub-domain of “diagnostic process”, we found a significant association with practice type (showing higher quality in multi-owner and group practices) and specific interest in periodontology (showing higher quality for members of the DGPARO).

**Table 4:** Association between practice-specific parameters (predictors) and information quality. Generalized linear modelling was performed to assess associations. Practice-specific parameters were entered simultaneously. Overall and domain-specific information quality (as outcomes) are given in the left column. Regression coefficients and 95% confidence intervals (in parentheses) are shown. Statistically significant associations are highlighted in bold

Outcome	Model fit (likelihood, *p*-value)	Practice type (ref: single owner)	Area (ref. rural)	Age of owner (ref. <50)	Specialized (ref: yes)
Technical/ functional aspects	7.39, *p* = 0.29	0.18 (−6.93/7.28)	−8.30 (−22.6/6.00)	−5.05 (−12.30/2.19)	1.69 (−5.98/9.36)
Generic quality and risk of bias	5.78, *p* = 0.45	0.48 (−5.24/6.20)	−1.00 (−12.5/10.5)	−3.13 (−8.95/2.70)	2.07 (−4.10/8.25)
Etiologic factors for periodontitis	1.83, *p* = 0.94	8.08 (−5.81/21.9)	0.57 (−27.4/28.6)	2.48 (−11.7/16.7)	1.60 (−13.4/16.6)
Prognostic factors	5.79, *p* = 0.44	5.24 (−2.88/13.4)	7.11 (−9.24/23.5)	0.23 (−8.06/8.51)	−6.58 (−15.4/2.19)
Diagnostic process	16.6, *p* = 0.01	**0.75 (0.44/1.06)**	−0.19 (−0.82/0.44)	−0.05 (−0.31/0.21)	**−0.35 (−0.61/−0.09)**
Treatment stages	6.40, *p* = 0.38	6.44 (−14.4/27.1)	−15.9 (−57.7/25.9)	14.3 (−6.90/35.4)	−9.44 (−31.9/12.9)
Tooth retention or removal	5.57, *p* = 0.47	−0.65 (−2.13/0.82)	0.84 (−2.13/3.81)	−0.28 (−1.78/1.23)	−1.18 (−2.77/0.42)
Total	6.41; *p* = 0.38	3.46 (−2.34/9.27)	−1.77 (−13.4/9.92)	−0.53 (−6.44/5.39)	−2.19 (−8.46/4.08)

## Discussion

The internet is an increasingly relevant source of health information for patients, mainly as (a) patients are increasingly engaged in their health and (b) seeking such information online is easy and often comes at no costs at all. It is likely that such information will have an impact both on the relationship between patient and dentist [10] and subsequent treatment decisions [11], with patients understanding their disease, but also its prevention, diagnosis and management. Information presented by dentists on their website are one major source for patients seeking dental health information, and it is conceivable that any information presented there has been placed with the agreement of the practice owner(s). Consequently, a number of relevant questions emerge; how reliable is the website content?, how current?, how helpful for patients’ decision making? Given that periodontitis is highly prevalent, being the second-most burdening dental disease [12, 13], it could be expected that on most dentists’ websites, some information on this disease was available.

Previous research in this direction has found poor quality of information on websites mentioning periodontitis, mainly as the accountability of the owner for the website was low and risk of bias high. It was also found that dentists’ websites performed poorer compared with journalistic or professional health information websites, and that sites identified via Medline showed higher quality of information than dentists’ websites [8]. The present study assessed the quality of information not only using generic tools (as has been done before), but also using a range of disease-specific criteria. Moreover, we used two established tools (LIDA, DISCERN) for technical and functional assessment and the generic risk of bias assessment, while alternatives like the JAMA scoring system were not used. This, notably, might hamper comparisons of generic tool assessment findings across studies, which calls for a standard tool for this purpose to be agreed on. Our hypothesis was that there are significant differences in information quality depending on practice-specific parameters. This was not the case; we thus reject our hypothesis.

When looking into different domains, we found the technical aspects of most websites to be largely satisfactory; most websites were found accessible, easy to use and read, functional and current. Moreover, the aims of most German websites were clearly laid out and it was stated that a third party was responsible for the support of these websites. This was is in contrast to the previously mentioned study, which found that most websites using English as language had not provided such information [8]. In contrast to these technical aspects, the generic risk of bias (indicated by a clear identification of the source of any presented information, the considerations towards any uncertainty, the general benefits and risks of treating periodontitis or not, and the available treatment options) were reported suboptimally by most German websites. The lowest quality scores were found for periodontitis-specific information. While the aspects of plaque leading to attachment loss and further factors contributing to the periodontitis risk were somewhat described in most sites, the prognostic factors, the diagnostic steps and the treatment of periodontitis were only insufficiently reported or discussed. Information on the chance of retaining teeth and comparisons with the risks associated with implants were basically absent.

There are a number of possible reasons for these findings.

- First, it is conceivable that having a fully-informative website is something most dentists regard as not needed, for example as (a) maintaining currency in this regard is laborious, (b) informing patients online is seen not as dentists’ tasks, (c) assuming that patients do not choose their dentists based on website quality, but other factors, (d) providing such information is seen as unnecessarily reducing information advantages, making further discussion with patients more difficult. The latter has been shown before, with doctors’ perceiving appointments being less efficient and more difficult in case patients were primed by online information [10]. This fear, in turn, might not be unjustified, as patients seem to selectively process online health information (most likely as part of a coping strategy), which leads to a biased understanding of their own disease and subsequent decision making [14]. (e) A last possible reason why information on periodontitis was of poor quality could be the “standing” of periodontitis therapy in German dental practices. As indicated by the limited number of treatment claims made to the statutory insurance in Germany (which covers the vast majority of the population) for periodontal therapy, managing periodontitis is not a major part of daily general practice or a significant contributor to dentists’ income [15]. It is possible that providing information on other aspects (like orthodontics, implantology or prosthodontics) seems more relevant to dentists. Comparing our findings on information quality with those from endodontics, orthodontics or caries therapy, for example, shows that the quality yielded in these fields was largely moderate to high with regards to technical aspects and generic risk of bias (measured as well using LIDA and DISCERN) [16–18]; i.e. significantly better (especially with regards to risk of bias). Direct comparisons with regards to disease specific information are only limitedly possible (the information found on orthodontic retainers seemed to at least be moderate in most websites assessed by a recent study) [18].
- Second, the poor ratings received by most websites in our study might be due to too strict information criteria being applied by us. These criteria were developed based on a guideline; the presented criterion catalogue could thus be seen as gold standard, but might not be realistically expected on dentists’ websites.
- Third, it might be that German websites are less informative than websites from other countries (something which is unlikely given the poor quality of information described above for English-speaking websites) [8].
- Fourth, our search was rather unspecific, using standard search engines. Using more specific search terms or browsing via curated search engines (like Medline) might yield a set of websites with higher quality. However, it could be doubted that patients use very specific terms for such searches or regularly search Medline for health information. In this regard, it would be useful to assess not only internet content, but the information on health applications, which are increasingly popular.

We had expected that certain practice-specific parameters like setting (higher quality in websites from urban practices given a different, more information-seeking clientele), ownership status (higher quality in multi-owner practices given the costs for setting up and maintaining a website being distributed among owners), age of the lead dentist or owner (higher quality in practices with younger owners given them being more web-affine) and specialization status (higher quality in practices of DGPARO members or specialists given their focus in this direction and the practice website being one tool to market that status) impact on a website’s quality. However, none of these parameters showed any significant association with overall quality. Only the information quality on the process for diagnosing periodontitis was significantly higher in multi-owner, specialized compared with single-owner, non-specialized practices. It should be noted that non-significance should not be confused with non-difference, as the power of our study might have been insufficient. That was, as around half of the quality scores were given for technical/functional aspects and generic risk of bias, with most studies fairing relatively well in this domain. Consequently, quality differences in small subdomains like diagnostic process or treatment steps (where there was considerable heterogeneity) were hard to detect. Future studies should account for these aspects during their sample size calculation. Overall, however, it seems that practice-specific parameters play a lesser role than expected, with disease-specific information quality being generally rather poor.

This study has a number of limitations. First, our sample – while being based on a sample size estimation - was relatively small (with the discussed issues of limited power), but yielded by representative sampling. Second, only German websites had been assessed, allowing to make a statement on a rather large health market, but not permitting conclusions as to websites from other countries. Third and as mentioned above, we did not use normalized overall quality scores (with each domain being re-weighted, accounting for some domains involving more questions than other) but used a sum score, which is biased towards larger domains. Last, while we used two validated assessment instruments (LIDA and DISCERN), there was no such instrument available to evaluate periodontitis-specific information quality. Abstracting such tool from a guideline document (as done in this study) might introduce bias into our assessments. However, we have shown both inter- and intra-rater reliability to be largely moderate; we therefore assume that using the developed checklist should yield reproducible results. There is, however, a need to develop an agreed standard for what websites on periodontitis should report on; such standard should also consider the efforts for maintaining the websites.

Future studies should assess how websites are produced and perceived by dentists; that is, what aims dentists have when setting up a website, which aspects they value highly, and which difficulties they encounter when developing and maintaining the website. It is also relevant to elucidate which impact the use of dentists’ website has on patients, for example with regards to their perception if teeth can be retained long-term despite periodontitis (something which many studies show) [19–21] or if removing and replacing teeth should be preferred instead. Moreover, there is the need to assess which patients access such internet content, i.e. is the target population of the information really the consumer population? As mentioned, national or supranational organizations could aim to develop a “master information set” which lays out what minimum information should be displayed on websites of dentists. The issue of information currency and possible automatization of website updates should be researched.

## Conclusions

Within the limitations of this study, most German dentists’ websites on periodontitis are not fully trustworthy. While these websites have sufficient technical quality, the risk of bias is rather high, and information on relevant aspects with regards to disease prevention, diagnosis and management are not or insufficiently considered. Patients should be aware of the resulting low quality of information and should seek such information elsewhere. Dentists’ should understand such findings as encouragement for providing better health information, and professional or regulatory bodies should develop ways of assisting the improvement and maintenance of the information quality from dentists’ websites.

## References

[1] Miniwatts Marketing Group (2016) Internet Users and 2016 Population. Available at: http://www.internetworldstats.com/stats.htm. Accessed 25 Jan 2017.

[2] Rainie L, Fox S (2000) The online health care revolution. Available at: http://www.pewinternet.org/2000/11/26/the-online-health-care-revolution/. Accessed 25 Jan 2017.

[3] Eysenbach G, Köhler C (2002). How do consumers search for and appraise health information on the world wide web? Qualitative study using focus groups, usability tests, and in-depth interviews. BMJ.

[4] Minervation. LIDA tool. 2016. Available at: http://www.minervation.com/wp-content/uploads/2011/04/Minervation-LIDA-instrument-v1-2.pdf. Accessed 27 July 2017.

[5] Charnock D, Shepperd S, Needham G, Gann R (1999). DISCERN: an instrument for judging the quality of written consumer health information on treatment choices. J Epidemiol Community Health.

[6] Keogh CJ, McHugh SM, Clarke Moloney M, Hannigan A, Healy DA, Burke PE, Kavanagh EG, Grace PA, Walsh SR (2014). Assessing the quality of online information for patients with carotid disease. Int J Surg (London, England).

[7] Muthukumarasamy S, Osmani Z, Sharpe A, England RJA (2012). Quality of information available on the world wide web for patients undergoing thyroidectomy: review. J Laryngol Otol.

[8] Bizzi I, Ghezzi P, Paudyal P (2017). Health information quality of websites on periodontology. J Clin Periodontol.

[9] Mombelli A, Schmid J, Walter C, Wetzel A (2014). QUALITÄTSLEITLINIEN: Parodontologie. Swiss Dent J.

[10] Murray E, Lo B, Pollack L, Donelan K, Catania J, Lee K, Zapert K, Turner R (2003). The impact of health information on the internet on health care and the physician-patient relationship: national U.S. survey among 1.050 U.S. physicians. J Med Internet Res.

[11] Andreassen HK, Bujnowska-Fedak MM, Chronaki CE, Dumitru RC, Pudule I, Santana S, Voss H, Wynn R (2007). European citizens’ use of E-health services: a study of seven countries. BMC Public Health.

[12] Kassebaum NJ, Bernabe E, Dahiya M, Bhandari B, Murray CJ, Marcenes W (2014). Global burden of severe periodontitis in 1990-2010: a systematic review and meta-regression. J Dent Res.

[13] Marcenes W, Kassebaum NJ, Bernabé E, Flaxman A, Naghavi M, Lopez A, Murray CJL (2013). Global burden of oral conditions in 1990-2010: a systematic analysis. J Dent Res.

[14] Sassenberg K, Greving H (2016). Internet searching about disease elicits a positive perception of own health when severity of illness is high: a longitudinal questionnaire study. J Med Internet Res.

[15] KZBV Jahrbuch 2015. Avaible at: http://www.kzbv.de/kzbv-jahrbuch-2015-2.media.b58d83c8c7fb6f73b35563fab351cb72.pdf. Accessed 27 July 2017.

[16] Blizniuk A, Furukawa S, Ueno M, Kawaguchi Y (2016). Evaluation of English websites on dental caries by using consumer evaluation tools. Oral Health Prev Dent.

[17] Rossi-Fedele G, Musu D, Cotti E, Doğramacı EJ (2016). Root canal treatment versus single-tooth implant: a systematic review of internet content. J Endod.

[18] Dogramaci EJ, Rossi-Fedele G (2016). The quality of information on the internet on orthodontic retainer wear: a cross-sectional study. J Orthod.

[19] Fardal O, Johannessen AC, Linden GJ (2004). Tooth loss during maintenance following periodontal treatment in a periodontal practice in Norway. J Clin Periodontol.

[20] McLeod DE, Lainson PA, Spivey JD (1998). The predictability of periodontal treatment as measured by tooth loss: a retrospective study. Quintessence Int (Berlin, Germany : 1985).

[21] Graetz C, Plaumann A, Schlattmann P, Kahl M, Springer C, Salzer S, Gomer K, Dorfer C, Schwendicke F (2017). Long-term tooth retention in chronic periodontitis - results after 18 years of a conservative periodontal treatment regimen in a university setting. J Clin Periodontol.

